# Transplantation of human microbiota into conventional mice durably reshapes the gut microbiota

**DOI:** 10.1038/s41598-018-25300-3

**Published:** 2018-05-01

**Authors:** Laura Wrzosek, Dragos Ciocan, Patrick Borentain, Madeleine Spatz, Virginie Puchois, Cindy Hugot, Gladys Ferrere, Camille Mayeur, Gabriel Perlemuter, Anne-Marie Cassard

**Affiliations:** 1 INSERM U996, Inflammation Chemokines and Immunopathology, Faculté de Médecine-Univ Paris-Sud, Université Paris-Saclay, Clamart, France; 2 Service d’Hépato-Gastroentérologie, Hôpital de la Timone, Marseille, France; 3 INRA, UMR 1319 MICALIS, AgroParisTech, Jouy-en-Josas, France; 4 AP-HP, Hepatogastroenterology and Nutrition, Hôpital Antoine-Béclère, Clamart, France

## Abstract

Human microbiota-associated (HMA) mice are an important model to study the relationship between liver diseases and intestinal microbiota. We describe a new method to humanize conventional mice based on bowel cleansing with polyethylene glycol followed by fecal microbiota transplantation (FMT) from a human donor. Four successive bowel cleansings were sufficient to empty the intestine and decrease the microbiota by 90%. We then compared four different strategies based on the frequency of FMT over four weeks: (1) twice a week; (2) once a week; (3) two FMTs; (4) one FMT. We were able to transfer human bacteria to mice, irrespective of the strategy used. We detected human bacteria after four weeks, even if only one FMT was performed, but there was a shift of the microbiota over time. FMT twice a week for four weeks was too frequent and perturbed the stability of the newly formed ecosystem. FMT once a week appears to be the best compromise as it allowed engraftment of *Faecalibacterium*, and a higher diversity of bacteria belonging to the Bacteroidales order. Our easy to establish HMA mouse model could be used as an alternative to classical HMA mice to study the relationship between the liver and the microbiota.

## Introduction

The gastrointestinal tract of mammals is colonized by a complex and dense bacterial community with a genome 150-fold larger than the human genome and a unique metabolic repertoire^1,2^. It is now well-established that the intestinal microbiota (IM) is a key player in intestinal and physiological homeostasis, immunity, and energy metabolism. Altered composition (dysbiosis) and functionality of the IM has been described in several human diseases, such as obesity, type 2 diabetes, autoimmune diseases, cardiovascular disease^3^, inflammatory bowel disease^4^, cancer^5^, liver diseases^6,7^, and psychiatric diseases^8^. Many studies have shown correlations between a specific profile of the IM and diseases. However, such data do not establish a causal link. A disease may modify the IM and conversely, the IM may trigger or aggravate a disease.

Human microbiota-associated (HMA) mice are an important tool to study the relationship between disease and the IM^9^. Such models generally consist of germ-free mice colonized with an IM recovered from human donors (fresh or frozen fecal samples). This leads to an IM in the recipient mice with a level of diversity similar to that of the human donor and high engraftment of the human IM^10^. This model has been used to show a causal relationship between IM and several diseases, including diet-induced obesity and non-alcoholic fatty liver diseases (NAFLD)^10,11^. However, HMA mice obtained using germ-free mice have many biological and technical limitations. Indeed, the IM is necessary for complete maturation of the gut^12^ and the host immune system^13,14^. Moreover, germ-free mice have atrophic Peyer’s patches, fewer intestinal IgA-secreting plasma cells, and lower B and T cell content than conventional mice^15^. Studies have shown that IM interactions during early life is a critical determinant for immune and metabolic function and alterations of microbiota during this developmental window can have long-term consequences, such as increased morbidity in models of IBD and allergic asthma^16,17^. There are major differences between the properties of the mucus of germ-free mice, which is penetrable to bacteria, and conventional mice. The IM is necessary to obtain a fully mature, sterile, mucus layer that is impenetrable to bacteria, which is fully formed by six weeks of age^18^. Moreover, breeding germ-free mice requires a suitable infrastructure, substantial financial resources, and most genetic mouse models are not available in a germ-free status^19^.

Other models of HMA mice have been developed using conventional mice and antibiotic treatment to deplete the resident IM and replace it with a human IM to overcome these limitations. Most of the studies have only shown the feasibly of these models^19,20^ and they have been seldom used in a disease context^21,22^. Instead, antibiotics were used to eradicate the endogenous IM to demonstrate its role in several pathological conditions^23,24^. HMA models using antibiotics should be used with caution, depending on the disease that is studied. For example, the use of antibiotics can be problematical when studying some diseases, as they may be improved in rodent models by the antibiotics themselves^23,25,26^. We have specifically focused on the role of the IM in alcoholic liver disease (ALD)^7,27^. One of the challenge of IM studies in ALD is to use HMA mice that do not rely on the use of germ-free mice or antibiotic treatment. Mucus plays an important role in ALD^27,28^ and it takes six weeks to have a mature mucus layer in HMA mice obtained using germ-free mice^18^, whereas the period of alcohol intake in ALD models is short^29^. In addition, antibiotics in ALD prevents liver injury^26^.

Here, we developed a new strategy based on conventional (*i*.*e*. not germ-free) mice, humanized by fecal microbiota transplantation (FMT), but without the use of antibiotics. We used polyethylene glycol (PEG) to perform bowel cleansing and dislodge the indigenous IM and then transplanted a human IM from a healthy donor. We suggest that our original model could be used as an alternative or a complement to classical HMA mice (*i*.*e*. germ-free mice or antibiotic treatment) to study the role of the IM, in particular in liver diseases, such as ALD.

## Results

### PEG cleansing decreases bacterial load in the gut

**Figure 1:**
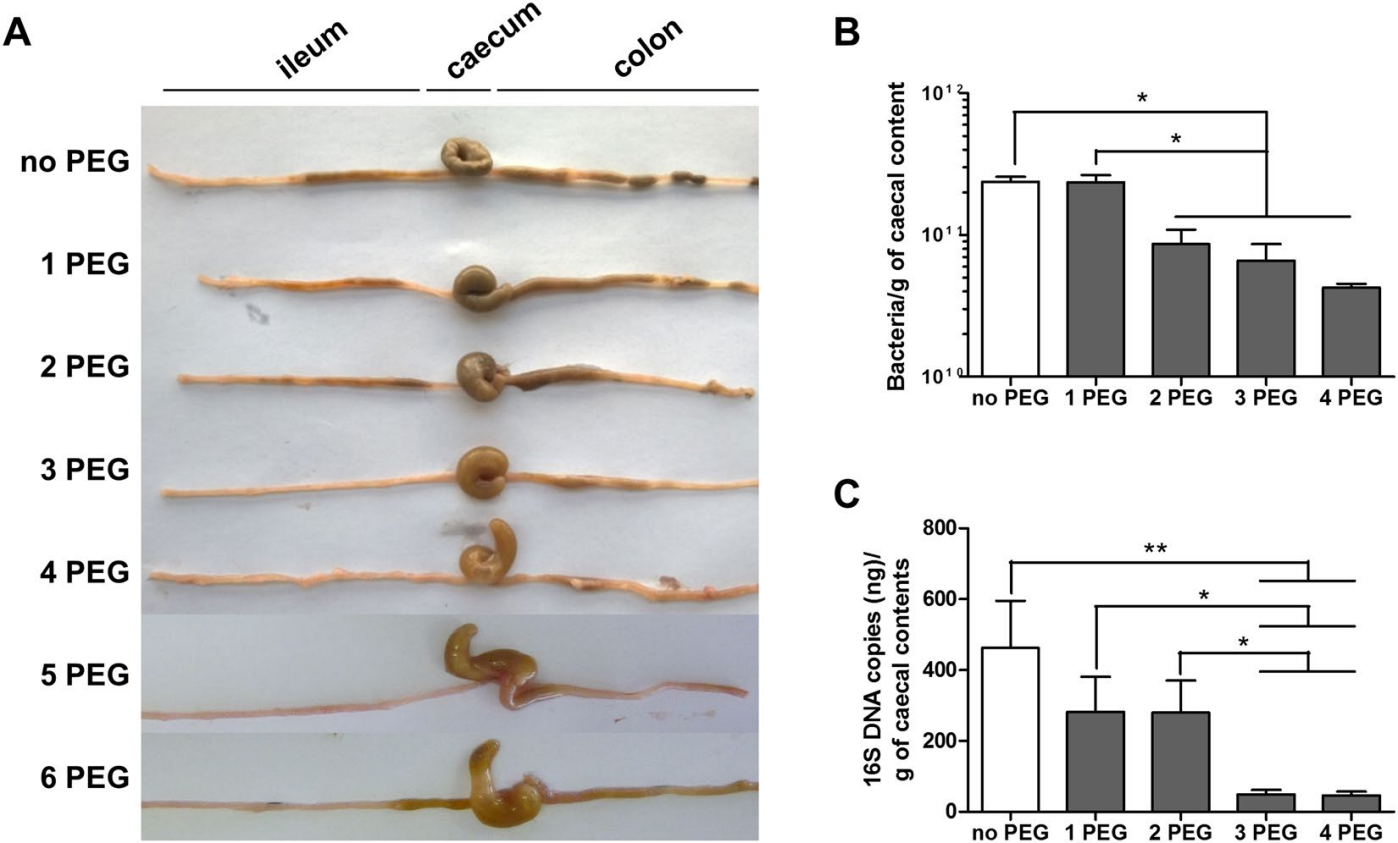
Effect of bowel cleansing with PEG on the microbiota. Mice were subjected to bowel cleansing with PEG solution by oral gavage. No PEG: no bowel cleansing; 1 PEG: mice receiving one bowel cleansing; 2 PEG: mice receiving two bowel cleansings; 3 PEG: mice receiving three bowel cleansings; 4 PEG: mice receiving four bowel cleansings; 5 PEG: mice receiving five bowel cleansings; 6 PEG: mice receiving six bowel cleansings. (**A**) Representative picture of the intestine of mice after bowel cleansing. (**B**) Quantification of luminal microbiota and (**C**) mucosal microbiota by Real-time qPCR analysis of the 16S rRNA gene. ^*^p < 0.05, ^**^p < 0.01.

We first aimed to determine the number of bowel cleansings that were necessary and sufficient to maximally empty the intestine of its contents. Mice received one to six bowel cleansings with PEG at 20-minute intervals. Bowel emptying four hours after PEG intake progressively improved from one to six cleansings with PEG (Fig. 1A). Intestinal contents were clearer and more liquid from four bowel cleansings. A plateau was reached after the fourth bowel cleansing and no additional effect was observed with five or six bowel cleansings. We then assessed the bacterial load by qPCR by 16S DNA gene targeting of all bacteria. We studied the luminal and mucosal IM (IM associated with the mucus layer). Bowel cleansing was associated with a significant one Log decrease (90% of the total bacteria) in the quantity of the luminal and mucosal IM (Fig. 1B and C), suggesting that four bowel cleansings were necessary and sufficient to empty the intestine and decrease bacterial load.

### Conditioning the feces before freezing has no major impact on IM diversity or composition

**Figure 2:**
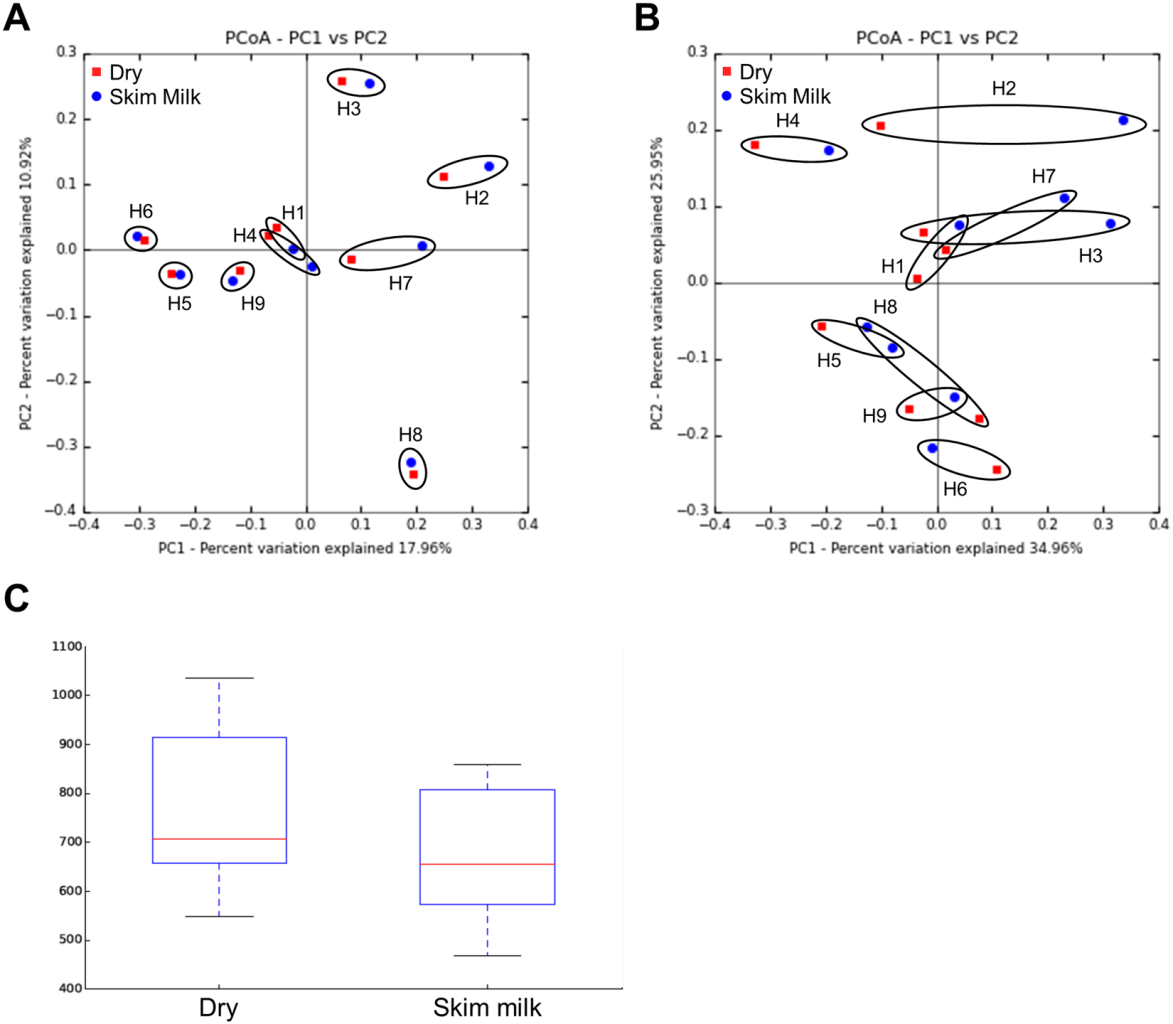
Preservation of the composition and structure of the intestinal microbiota following resuspension and freezing. PCoA plot showing the (**A**) unweighted UniFrac distance (p < 0.001) and (**B**) weighted UniFrac distance (p < 0.001). Red squares indicate freeze-dried feces samples and blue circles indicate frozen feces resuspended in BHI + skim milk media. Each encircled area corresponds to samples from the same human donor (H1 to H9). (**C**) Box plots showing microbiota diversity based on the OTUs observed (counts of unique OTUs).

The fecal samples of patients were processed and conditioned immediately after collection, before being frozen for a potential later transplantation into mice (for further FMT experiments in conventional mice, we used a single human donor). It consisted of suspending and diluting the feces in skim milk + BHI media. We assessed whether processing fecal samples from patients may alter IM composition, and thus alter the results of FMT. We compared the IM of human donors from freeze-dried or frozen diluted feces by deep sequencing before FMT. This molecular analysis was based on the 16S rRNA gene and not the culture of viable bacteria. Beta-diversity analysis using unweighted UniFrac PCoA (principal coordinate analysis) provides an overview of IM composition in terms of the presence/absence of bacteria (Fig. 2A), whereas weighted UniFrac PCoA describes the IM structure and integrates bacterial abundance (Fig. 2B). PCoA plots of unweighted and weighted UniFrac distances showed that IM composition and structure of freeze-dried feces clustered with that of frozen diluted feces for each human donor considered. Alpha-diversity analysis was similar between freeze-dried and frozen diluted feces showing that OTUs (operational taxonomic units) richness was not affected by processing of the samples (Fig. 2C). These results show that the processing and conditioning of feces had no major impact on the bacterial ecosystem relative to that of freeze-dried feces. Thus, mice transplanted with feces diluted in skim milk + BHI received a fecal sample during FMT with a composition similar to that of freeze-dried feces.

### FMT of the human IM can change the IM in conventional mice

**Figure 3:**
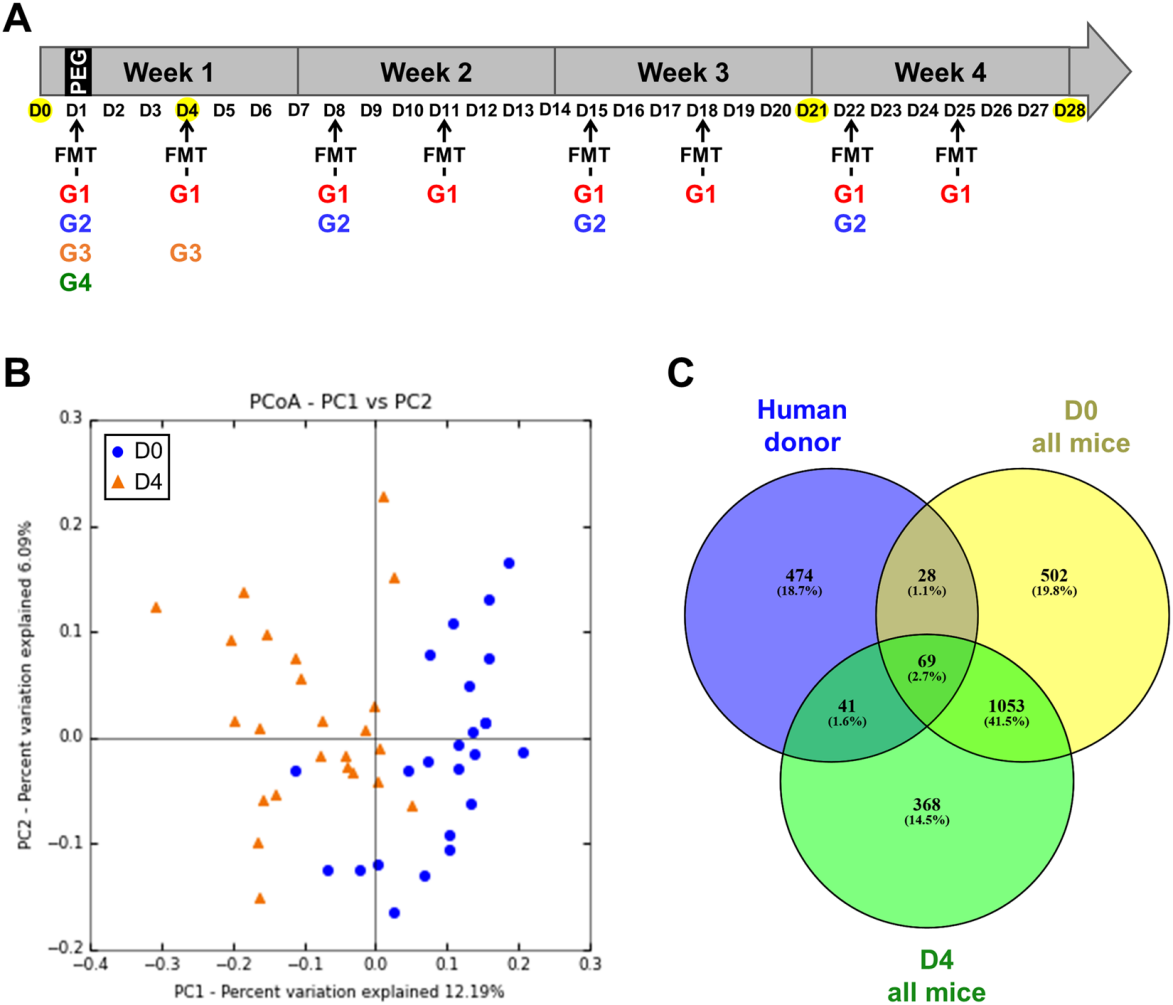
Modification of the murine microbiota following human FMT. (**A**) Timeline showing the human intestinal microbiota inoculation scheme in conventional mice. Arrows indicate each microbiota inoculation by oral gavage and yellow circles indicate collection of feces samples for all mice of the protocol. (**B**) PCoA plot showing the unweighted UniFrac distance (p < 0.001). Blue circles correspond to the microbiota at D0 and orange triangles to the microbiota at D4. (**C**) Venn diagram based on OTUs distribution between the human donor and all the mice at D0 or D4.

We aimed to further determine the best strategy of FMT to transfer the human IM into conventional mice following bowel cleansing. All mice received four doses of PEG to dislodge their indigenous IM on the first day (D1) of the experiments only (Fig. 3A). This was followed four hours later by FMT from a single healthy human donor. Mice were then divided into four groups: the first group of mice (G1) received FMT twice a week for four weeks; the second (G2) FMT once a week; the third (G3) FMT twice the first week, and the fourth (G4) only one FMT the first week on the first day of the experiment (D1). The G3 and G4 groups did not receive any other FMT (Fig. 3A).

The IM collected at D0 represents the indigenous IM of the mice. The IM was collected at D4, after all the mice received one FMT and just before another FMT was performed, depending on the group. We pooled all the mice at D0 (mice with indigenous IM) and all those at D4 (HMA mice) and analyzed the IM. Unweighted UniFrac PCoA showed that the IM of mice was modified by FMT between D0 and D4 after FMT (Fig. 3B). The Venn diagram plot of the sequenced IM depicts the distribution of OTUs shared by the human donor and mice at D4 and D0 (Fig. 3C). We then focused on the OTUs that were only shared by the human donor and mice at D4 and absent from the indigenous IM of the mice (D0). Indeed, these OTUs correspond to those successfully transplanted from human to mouse following FMT. Forty-one OTUs were absent from mice at D0 and shared by the human donor and the mice at D4. They mostly belonged to the genus *Bifidobacterium*, *Collinsella*, *Odoribacter*, *Bacteroides* (*B*. *fragilis* and *B*. *ovatus* species), *Parabacteroides*, and *Roseburia* and the Lachnospiraceae family. Only a few OTUs belonging to the phylum Proteobacteria were transferred at D4 (*Sutterella* and *Bilophila* genus) (Supplemental Table 1). These OTUs specifically originate from the human IM and were transferred to the mice at an early time point. Thus, PEG cleansing was sufficient to allow the engraftment of human species in conventional mice.

### Human bacteria are detected in recipient mice four weeks after FMT, regardless of the FMT strategy

We next assessed whether the OTUs from the human donor were maintained over time in the mice (*i*.*e*. after D4), and whether new OTUs from the human donor were transferred to the mice, depending on the FMT strategy.

**Figure 4:**
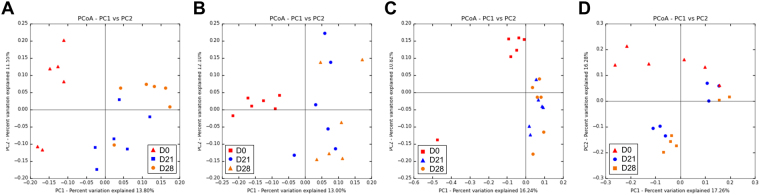
Evolution of the microbiota at the end of the experiment depending on the FMT strategy. Red symbols: microbiota at D0; blue symbols: microbiota at D21; orange symbols: microbiota at D28. PCoA plot showing the unweighted UniFrac distance in (**A**) Group 1 (p < 0.001); (**B**) Group 2 (p < 0.001); (**C**) Group 3 (p < 0.001); and (**D**) Group 4 (p < 0.001).

We compared the different FMT strategies on IM composition of the recipient mice according to the frequency of FMT over the four weeks (G1, G2, G3 or G4). Beta-diversity analysis using unweighted UniFrac distance PCoA plots showed that the IM of mice at the end of the experiment (D28) was modified relative to the beginning of the experiment (D0), irrespective of the FMT strategy (Fig. 4A,B,C,D). The IM was even modified at D21 and D28 in group 4, which received only one FMT. This result suggests that only one FMT is sufficient to durably modify the IM. Moreover, the transfer remained stable four weeks after FMT (Fig. 4D). However, there was individual variability in IM composition associated with the housing of mice in two different cages. The IM reached a steady state by the end of the experiment as the IM at D21 and D28 clustered together in all the groups.

However, engraftment of the human IM in recipient mice varied depending on the frequency of FMT (Supplemental Tables 2, 3, 4, 5). We analyzed the OTUs tables to identify specific bacteria from the human IM that were transferred to mice following FMT. We focused on the specific OTUs from the human IM which were absent from mice at D0 and detected after FMT (HMA mice) at D21 and D28. Thus, we were able to discriminate between OTUs shared by the human donor and mice following FMT because we excluded OTUs specific to human or mice and OTUs shared by the human donor and mice before FMT. The IM of the mice was modified in all groups, but not in the same manner, depending on the FMT strategy (Supplemental Tables 2, 3, 4, 5). For example, we detected *Parabacteroides distasonis* in all groups, irrespective of the FMT strategy. *Faecalibacterium prausnitzii* was only detected in groups of mice receiving repeated rounds of FMT, twice a week or once a week (G1 and G2), and this species was never detected before D28. In contrast, we did not detect *Bifidobacterium* in G1 and G2 (FMT twice a week and once a week, respectively) but detected it in G3 and G4 (FMT the first week).

## Discussion

Here, we describe a new method to humanize conventional mice using bowel cleansing with PEG, without antibiotic treatment, followed by FMT. We compared several FMT strategies, based on the frequency of transfer and followed the evolution of the IM in recipient mice for four weeks.

We used frozen feces from a healthy human donor preserved in a mix of BHI and skim milk media supplemented with cysteine to perform FMT. Skim milk enhances the preservation of thawed bacterial stocks stored at −80 °C and cysteine is used as a medium reducer to preserve anaerobic bacteria^30^. We showed that the IM can be preserved, irrespective of the method used to process the feces (freeze-dried or frozen in BHI/skim milk). This is in accordance with published results showing that the fecal microbial community remained stable in samples stored for a long period at −80 °C and shared more identity with its host of origin than any other sample^31,32^.

In our model, we observed a decrease (*i*.*e*. one Log) in the bacterial load after bowel cleansing. Jalanka *et al*. described a similar decrease in patients undergoing a bowel cleansing also using macrogol^33^. PEG alone can lead to a similar decrease in the quantity of IM bacteria as a combination of antibiotics. Indeed, a decrease of up to four Logs of the 16S DNA using a combination of vancomycin, metronidazole, and neomycin or one Log using ampicillin or vancomycin alone^34^ or a combination of vancomycin and imipenem^35^ were reported for antibiotic-treated rodents. Antibiotics are often used to mimic a germ-free status, but this is not the case, and they may affect many biological pathways. Antibiotics may lead to downregulation of T-lymphocyte numbers in the gut^36^. Metronidazole affects goblet-cell function by decreasing Muc2 production, resulting in thinning of the inner protective mucus layer^37^ and it is known that mucus plays an important role in several diseases, such as ALD^7,27,28,38^ or inflammatory bowel diseases^39^. The challenge to create HMA mice using conventional mice consists of eradicating the indigenous IM to allow engraftment of the human IM. Even if luminal and mucosal IM were not drastically decreased after bowel cleansing, we anticipated that the spatial distribution of the IM may be considerably disrupted, offering a niche for the exogenous human IM. Indeed, this aspect may be more important than simply decreasing the quantity of bacteria *per se*. This is in accordance with the results of Manichanh *et al*. who suggested that the establishment of an exogenous IM is likely conditioned more by the alteration of gut microbiome homeostasis caused by antibiotics than the decrease in bacterial load *per se*^35^.

Imperfect transfer of the human donor IM has already been described for various models of HMA mice (*i*.*e*. germ-free mice or antibiotic-treated conventional mice), with selective enrichment of Bacteroidetes over the Firmicutes phylum^10,19,20,40^. Moreover, lowering bacterial load by antibiotics prior to FMT has not always been associated with better transfer of the donor IM^35^. A study that compared the efficacy of various gut preparation procedures commonly used before FMT in patients, such as antibiotic treatment, bowel cleansing, and no pre-treatment^41^, led to the conclusion that the best IM engraftment was obtained following antibiotic treatment. Nevertheless, the endpoint was the Bray-Curtis dissimilarities between groups. This study did not focus on more detailed analyses of the transferred OTUs. Moreover, the Bray-Curtis dissimilarity is frequently used to quantify differences between samples based only on abundance and does not consider the specific OTUs that are transferred.

In our model, only a part of the human IM was transferred to mice, irrespective of the FMT strategy. Although in the present study we did not compared our model to a germ-free HMA mice model and we only transferred a small proportion of the IM, we have already shown that such a transfer was sufficient to modify the phenotype of the recipient mice^7^ and that it could be an alternative to germ-free HMA mice. In this previous study, we demonstrate that the individual sensitivity to alcohol is driven by IM^7^. Germ-free HMA mice with IM from an alcoholic patient with severe ALD developed more important alcoholic liver lesions than germ-free HMA mice with IM from an alcoholic patient without liver lesions. Similar results were obtained in conventional HMA that received human IM after bowel cleansing with PEG^7^. Therefore, these FMT experiments in two different models of HMA mice (using germ-free or conventional mice with bowel cleansing with PEG) leading to similar results suggest that bowel cleansing with PEG before FMT in conventional mice can be an efficient alternative to germ-free mice, at least in ALD.

Finally, we were able to transfer human OTUs into recipient mice, irrespective of the strategy used. We detected human OTUs four weeks later, even after only one FMT. However, with just one FMT, we observed a cage effect on IM composition, probably because of a shift over time due to coprophagy and lower stability of the IM. Some OTUs required a low frequency of FMT to become established in recipient mice, such as *Bifidobacteria*. This is in accordance with a study that showed that the host microbiota can sustain configurational changes after exposure to transient bacteria^42^. Repeated FMT at very short intervals may disturb the newly established ecosystem and impair its equilibrium. However, some bacteria (*e*.*g*. *Faecalibacterium*) required repeated FMT to be transferred to mice. Thus, the best FMT strategy may depend on the disease and the type of bacteria to be studied. The strategy using solely one FMT should not be used, as there is a cage-dependent shift of the IM over time. In contrast, FMT twice a week for several weeks can affect the engraftment and perturb the stability of the newly formed ecosystem. FMT twice during the first week allowed engraftment of sub-dominant bacteria, such as *Bifidobacteria*, often used in probiotics. FMT once a week during the whole protocol may be the best compromise, as it allowed the engraftment of dominant bacteria, such as *Faecalibacterium*, which was not detected without recurrent FMT, and that of a higher diversity in the Bacteroidales order than FMT twice during the first week.

Our study has some limitations. We did not compare our strategy with the other models (germ-free or antibiotic treatment). Nevertheless, we have already shown the possibility to transfer liver sensitivity to alcohol in both germ-free mice and the present model^7^. Currently, there is no consensus on animal models of FMT from human donors and researchers should choose the model based on the disease studied. All available models, including ours, have their own advantages and disadvantages. It is important to be aware of them and choose the best model depending on the goal of the study. Most studies on HMA mice have focused specifically on bacteria shared by the IM of human donors and recipient mice, including ours. Although this is an important feature, FMT includes the transfer of more than bacteria. Viruses, archaea, and fungi are also components of feces and may affect recipient biology^43^, as it was recently shown that fungi are involved in the pathophysiology of ALD and cirrhosis^44,45^.

In conclusion, we describe a novel and powerful model, consisting of FMT into conventional recipient mice without the use of antibiotics. This model is easy to use and is an inexpensive method to perform FMT from humans to mice. Its usefulness has already been established in ALD studies. Our model will allow researchers to circumvent the use of germ-free mice and the associated logistical burden.

## Methods

### Collection and freezing of feces from human donors

Feces from human donors were recovered and immediately stored at 4 °C in an anaerobiosis generator (Genbox, Biomérieux, Capronne, France) to favor the preservation of anaerobic bacteria. All donors gave their informed consent^7^. These samples were used to study the impact of the feces conditioning before freezing on IM diversity and composition.

Among all these feces from human donors, the feces from a healthy 26-year-old woman with a BMI (body mass index) of 20.6, no ongoing disease, and no drug use in the previous three months were stored as described above and used for FMT into conventional mice two months after freezing. The donor gave her informed consent.

All samples were processed within 24 h and either freeze-dried at −80 °C for DNA extraction or resuspended, aliquoted, and frozen at −80 °C for FMT. For the latter, feces were rapidly diluted 100-fold in BHI (Brain Heart Infusion, Becton Dickinson) supplemented with 0.5 mg/ml L-cysteine (Sigma-Aldrich, St-Louis, MO, USA) and 20% skim milk (Becton Dickinson) (vol/vol) and stored in aliquots at −80 °C. This ready-to-use fecal preparation was administrated to mice for FMT. The BHI and 20% skim milk were sterilized according to the manufacturers’ recommendations and stored at 4 °C until use. Just before processing the feces, the BHI medium and the skim milk medium were boiled for 30 min and allowed to cool. We checked the viability of the bacteria in the FMT suspension.

This study received CPP (Comité de Protection des Personnes, Project ID RCB 2009-A00956-51) approval from the Ile de France VII ethics committee (Bicêtre Hospital, 94270 le Kremlin-Bicêtre, France). All methods were performed in accordance with the relevant local guidelines and regulations and all patients provided written informed consent for participation in the study.

### Mice

Eight-week-old female C57BL/6J mice were purchased from Janvier laboratory (Le Genest, France). The animals were kept in humidity and temperature-controlled rooms, on a 12-hour light-dark cycle, and fed a standard diet (Altromin 1310, Genestil, Royancourt, France). All experimental protocols were validated by the local ethics committee, CEEA26 and the “Ministère de la Recherche” (APAFIS#4255-20 1 6080812089425 v1). All methods were performed in accordance with the relevant local guidelines and regulations.

### Bowel cleansing procedure

We first determined the best procedure to empty the digestive contents of the mice. Seven groups of mice (four mice/group) were subjected, or not, to bowel cleansing by oral-gastric gavage with PEG (polyethylene glycol, Macrogol 4000, Fortrans, Ipsen Pharma, France). The day before bowel cleansing with PEG, we recovered feces from all mice and immediately froze them at −80 °C for further DNA extraction and IM analysis.

All groups of mice were fasted 1 h before the beginning of bowel cleansing (free access to water). Mice are coprophagous. Thus, they were placed in clean cages and subjected 1 h later to bowel cleansing by oral-gastric gavage with 200 µl PEG at 425 g/l. Some groups of mice received several oral-gastric gavages with PEG at 20-minute intervals. The “1 PEG” group received one round of oral-gastric gavage. The “2 PEG”, “3 PEG”, “4 PEG”, “5 PEG”, and “6 PEG” groups received two, three, four, five, or six rounds of oral-gastric gavage, respectively. The control group was not subjected to bowel cleansing, but received 200 µl water. Mice were euthanized by cervical dislocation 4 h after the end of bowel cleansing. Intestinal and caecal contents were recovered for each mouse. The colons were opened longitudinally and the fecal pellets carefully removed. The colons were then scraped to recover the mucosal microbiota and stored at −80 °C until DNA extraction and IM analysis.

### Human microbiota transplantation into conventional mice

We compared different strategies based on varying the frequency of FMT to ensure and maintain the implantation of the human IM into conventional mice. This experiment was carried out over four weeks and 24 mice were divided into four groups (six mice/group).

Group 1 (G1) included mice that received FMT twice a week for four weeks, group 2 (G2) received FMT once a week for four weeks, group 3 (G3) received two rounds of FMT the first week and then nothing, and group 4 (G4) received a FMT during the first week and then nothing (Fig. 2C).

At day 1, mice were subjected to bowel cleansing by oral gavage with four doses of 200 µl PEG at 425 g/l at 20-min intervals as already described. Four hours later, mice received the transplant by oral gastric gavage with 200 μl resuspended feces from the human healthy donor, prepared as described above. Mice were then allowed free access to food and water. Bowel cleansing was only performed on day 1. The feces were regularly recovered throughout the four weeks of the experiment (D0, D4, D21 and D28) and immediately stored at −80 °C until processing for DNA extraction and 16S deep sequencing. At the end of the experiment, mice were euthanized by cervical dislocation. Caecal contents were recovered for each mouse.

### DNA extraction from feces, caecal contents, and mucosal microbiota

Feces, caecal contents, or mucosal microbiota were collected from each mouse and immediately stored at −80 °C. Total DNA was extracted from 150 mg feces, 100 mg caecal contents, or 50 mg colonic mucosa as previously described^46^, with modifications^12^. Briefly, after nucleic acid precipitation with isopropanol, DNA suspensions were incubated overnight at 4 °C and centrifuged at 20,000 × *g* for 30 min. The supernatants were transferred to 2-ml tubes with 4 µl RNase (RNase A, 10 mg/ml; EN0531; Fermentas, Villebon sur Yvette, France) and incubated at 37 °C for 30 min. Nucleic acids were precipitated with 1 ml absolute ethanol and 50 µl 3 M sodium acetate and centrifuged at 20,000 × *g* for 5 min. The pellets were washed with 70% ethanol and centrifuged at 20,000 × *g* for 3 min. The washing step was repeated three times. Finally, the DNA pellets were dried at room temperature for 2 h and resuspended in 100 µl Tris-EDTA buffer. DNA suspensions were stored at −20 °C for real-time quantitative PCR (qPCR) analysis of the 16 S ribosomal genes or 16 S deep sequencing with Illumina MiSeq technology.

### Real-time qPCR analysis of bacterial 16S rRNA genes

The primers designed to detect all bacteria were based on 16S rDNA gene sequences: forward ACTCCTACGGGAGGCAGCAGT and reverse ATTACCGCGGCTGCTGGC. Detection was achieved with a Light Cycler 480 (Roche Diagnostics, Basel, Switzerland) using the LC FastStart DNA Master SYBR Green I kit (Roche Diagnostics) with a primer concentration of 10 μM and annealing temperature of 65 °C. The cycle threshold of each sample was then compared to a standard curve, performed in duplicate, made by diluting genomic DNA from an *Escherichia coli* culture. The data are expressed as nanograms of bacterial DNA per gram of caecal content.

### Analysis of the intestinal microbiota by 16S Ribosomal RNA sequencing

The composition of the microbiota was analyzed using Illumina MiSeq technology targeting the 16S ribosomal DNA V3-V4 region in paired-end modus (2 × 300 base pair) (GenoToul, Toulouse). PCR was performed to prepare amplicons using V3-V4 oligonucleotides (PCR1F_460: 5′ CTTTCCCTACACGACGCTCTTCCGATCTACGGRAGGCAGCAG 3′, PCR1R_460: 5′ GGAGTTCAGACGTGTGCTCTTCCGATCTTACCAGGGTATCTAATCCT 3′). Amplicon quality was verified by gel electrophoresis and they were sent to the GenoToul platform for sequencing.

The resulting paired reads were assembled using PANDAseq v 2.7 to generate an amplicon size of 450 base pairs^47^. Reads were demultiplexed and processed using the quantitative insights into microbial ecology (QIIME v1.9.0) pipeline and the default parameters of QIIME^48^. Chimeric sequences were identified *de novo*, reference based, and then removed using usearch61^49^. The non-chimeric sequences were then clustered into operational taxonomic units (OTUs) at 97.0% sequence similarity using a closed reference-based picking approach with UCLUST software against the Greengenes database 13_8 of bacterial 16S rDNA sequences^50^. When full assignment at the genus or species taxonomic level was not possible, g_ or s_ alone was used to designate genus or species, respectively. The mean number of quality-controlled reads was 28,675 ± 10,173 (mean ± SD) per mouse. After rarefaction at 6,000 reads per sample, bacterial alpha diversity was estimated using the Shannon index. OTUs with a prevalence < 5% were removed from the analysis. Results are represented as the mean ± SEM. The Wilcoxon test was used to assess statistical significance of the bacterial composition between the samples. Associations were considered to be significant after a false-discovery rate (FDR) correction of the p-value (q < 0.05).

Beta diversity was assessed using weighted and unweighted UniFrac distances. The weighted Unifrac metric is weighted by the difference in the abundance of OTUs from each community, whereas unweighted Unifrac only considers the absence/presence of the OTUs providing different information. Both are phylogenetic beta diversity metrics. The link between the various groups of mice and bacterial microbial profiles was addressed by performing an ANOSIM test with 10,000 permutations on the beta diversity metrics described above.

### Statistical analyses

Results are represented as the mean ± SEM. Statistical analyses were performed using the Mann-Whitney and Kruskal-Wallis tests (Graphpad Prism, Graphpad Software Inc, La Jolla, California, USA); p < 0,05 was considered to be statistically significant. ^*^p < 0.05, ^**^p < 0.01, ^***^p < 0.001.

## Electronic supplementary material

Supplementary Table 1

Supplementary Table 2

Supplementary Table 3

Supplementary Table 4

Supplementary Table 5
